# Diagnostic stability of attention deficit hyperactivity disorder during healthcare transition

**DOI:** 10.1016/j.hctj.2024.100089

**Published:** 2024-12-18

**Authors:** Alberto Rodríguez-Quiroga, Cristina Bonilla Sanz, Miguel Ángel Álvarez-Mon, Fernando Mora Mínguez, Javier Quintero

**Affiliations:** a Psychiatry and Mental Health Department, Hospital Universitario Infanta Leonor, Avenida de la Gran Vía del Este 80, Madrid 20830, Spain; b Department of Legal Medicine & Psychiatry, Universidad Complutense, Spain; c Department of Medicine and Medical Specialties, Universidad de Alcalá, Madrid, Spain

**Keywords:** ADHD, Combined presentation, Predominantly inattentive presentation, Predominantly hyperactive/impulsive presentation

## Abstract

**Background and Aims:**

The belief that ADHD remitted in adulthood and the absence of specific criteria for its diagnosis in adults have led to discrepancies in estimating its persistence, hindering proper treatment. The objective of this study was to evaluate the persistence of diagnosis and subtypes of ADHD in patients transitioning to adulthood in a specialized setting using DSM-5 criteria.

**Material and Methods:**

Retrospective data were collected from 59 patients diagnosed with ADHD at the Hospital Universitario Infanta Leonor, Madrid, with symptom onset between 2 and 12 years of age. Subgroups were formed based on diagnosis and initial subtype stability, and descriptive and statistical analysis was performed using SPSS software.

**Results:**

The persistence rate was 93.2 %. Persistence was significantly associated with the need for specific follow-up at present, but not with gender, current age, or treatment. High percentages of comorbidity were found in both the persistent and remission groups. The initial inattentive subtype showed greater stability, with a preservation rate of 95.83 %. The combined subtype decreased more over time, with a preservation rate of 71.4 %. The diagnostic consistency for each subtype was high, although overall diagnostic concordance decreased slightly with age.

**Conclusions:**

ADHD stability using appropriate criteria is higher than previously described. Comprehensive follow-up is necessary regardless of the current diagnostic status, especially in the period of transition from adolescence to adulthood.

## Highlights

• ADHD persistence in a specialized Unit was higher than previously reported.
• Agreement between initial and current ADHD diagnoses slightly decreases with age.
• Comprehensive follow-up in ADHD cases helps identify and manage concurrent disorders.
• The inattentive ADHD presentation is the most stable over time.

## 1 Introduction

Attention Deficit Hyperactivity Disorder (ADHD) has been classically associated with childhood and adolescence, affecting a proportion of between 7.6 % and 11.3 % and between 5.6 % and 12.7 %, respectively.^1^ Its clinical manifestations begin in this stage, so it is widely conceptualized in this population. In adulthood, ADHD is estimated to have a prevalence of 2.5–6.7 %.^2^ This condition may be underdiagnosed and undertreated for several reasons.

For many years, it was believed that ADHD remitted in adulthood, although it could increase comorbidity with other disorders. However, studies now show that ADHD often persists into adulthood, though the reported rates vary widely (4–77 %), partly due to inconsistent definitions of remission. Recent studies suggest this persistence may be underestimated due to a lack of adult-specific diagnostic criteria in earlier DSM editions.^3^ One review estimates that 50–60 % of cases persist when using sensitive criteria.^4^

Incomplete remission is common, with residual symptoms that continue to impact daily life, particularly in those diagnosed in childhood.^5^ These individuals often struggle with fluctuating symptoms, leading to difficulties in professional, academic, and social spheres, as well as contributing to the potential development of comorbid conditions, such as mood disorders, anxiety, and substance use disorders, which can mask the executive dysfunction of ADHD and delay diagnosis and treatment.^6^

Another key issue complicating the management of ADHD is the transition from child and adolescent mental health services (CAMHS) to adult mental health services (AMHS), which is often poorly planned and executed.^7^ The lack of adequate planning for the transition from child to adult psychiatric care can hinder treatment outcomes.^8^ Many adolescents discontinue treatment during this critical period, which can negatively affect their development as they navigate educational, occupational, and personal transitions.^9^ Individuals with ADHD, like those with other mental health conditions, may face reduced support during the transition to adulthood.^10^ This gap in care underscores the need for more structured transitional protocols and long-term treatment strategies that address both ongoing ADHD symptoms and emerging adult-specific challenges.

The DSM-5 establishes three different presentations (previously described as subtypes) based on the predominant symptoms: hyperactive-impulsive, inattentive, and combined.^11^ The assessment of the stability of each subtype of ADHD is beginning to gain importance for several reasons. Brain maturity and the acquisition of strategies in adulthood cause the central symptoms of ADHD to evolve, primarily hyperactivity, which becomes less evident and transforms into feelings of internal restlessness and unease.^12^, ^13^ However, difficulty in maintaining attention, as well as executive dysfunction symptoms, persist over time.^14^ Identifying patterns of change and evolution of symptoms as different developmental stages are traversed provides valuable information about areas of functioning that may require more attention and support at any given time. This can be relevant both for accurate diagnosis and for long-term treatment planning.^15^ It is therefore important to detect the change in symptoms over time as a more accurate measure of the clinical course than that obtained only by considering changes in diagnosis, so that interventions and treatment approaches can be adapted to the individual needs of each subtype, and the quality of life of individuals affected by ADHD improves.

The present work aims to evaluate the evolution and persistence of the diagnosis of ADHD and the initially diagnosed subtype over time in a group of patients collected in a specialized consultation, from when the diagnosis was made in childhood using DSM-5 criteria. The secondary objective would be to assess the presence of comorbidities and addictions, as well as to identify possible factors related to stability.

## 2 Material and methods

### 2.1 Study type and sample

An observational, descriptive, and retrospective study was conducted. We collected data from patients diagnosed with ADHD in a specialized ADHD consultation Unit at the Infanta Leonor University Hospital in Madrid. This unit is part of a larger psychiatric service that focuses on the long-term management of ADHD, covering the critical transition period from childhood to adulthood. The unit offers comprehensive care for patients across developmental stages, making it ideal for studying the evolution of ADHD diagnoses and symptoms over time.

Patients were diagnosed in childhood using DSM-5 criteria and have remained in long-term follow-up care at the unit. This setting is particularly significant as it provides continuity of care during the transitional period from child and adolescent mental health services (CAMHS) to adult mental health services (AMHS). This period is often characterized by challenges, including treatment discontinuation, but the unit’s integrated structure supports a smoother transition by offering both pediatric and adult services within the same setting. As such, this allows for the assessment of how this continuity of care influences ADHD symptomatology, diagnosis stability, and treatment outcomes over time.

All data were collected retrospectively through a review of medical reports, which were subsequently anonymized and stored in a database using Excel software. The sample included patients aged between 2 and 12 years at the onset of symptoms, who had been diagnosed with ADHD in childhood at this unit. Patients whose symptoms began after the age of 12 or whose initial diagnosis did not correspond to ADHD were excluded from the study.

### 2.2 Study design and definitions

To clarify the timepoints for diagnosis, the initial diagnosis was established based on clinical evaluations during the patient's first visit to the ADHD consultation unit. The final diagnosis reflects the assessment conducted during the most recent clinical evaluation, which occurred at an average follow-up of 3.5 years (range: 1–6 years) after the initial diagnosis. At the time of the latest evaluation, participants had a mean age of 14.5 years, with an age range of 7–18 years. The ages at the time of the first evaluation ranged from 4 to 17 years, indicating a developmental span that includes early childhood through late adolescence.

Given the developmental changes that occur during adolescence, it is common practice to conduct multiple assessments for ADHD. This allows for monitoring symptom changes, determining the persistence of the diagnosis, and informing appropriate treatment decisions.

We evaluated the stability of both the ADHD diagnosis itself and its constituent subtypes, leading to the following groups:

Persistent ADHD and ADHD in remission, based on the presence or absence of an ADHD diagnosis in the most recent clinical evaluation.

Preserved initial subtype and non-preserved initial subtype, based on the presence or absence of the initially diagnosed subtype in the most recent evaluation.

The diagnostic criteria applied correspond to those specified in the DSM-5 for children or adults, as appropriate. In both cases, we evaluated the proportion of persistent cases and the association of other clinical variables with the presence or absence of stability in the diagnosis.

### 2.3 Studied variables

This study examined various variables to assess the stability of ADHD diagnoses and associated factors over time. The variables were categorized as follows:

#### 2.3.1 Demographic variables

##### 2.3.1.1 Age

Current age of participants at the time of the final evaluation.

##### 2.3.1.2 Gender

Categorical variable (male, female) indicating the sex of participants.

#### 2.3.2 Clinical variables

##### 2.3.2.1 Initial diagnosis

The diagnosis established at the time of the first visit to the ADHD consultation unit, classified into ADHD subtypes as per DSM-5 criteria (e.g., combined type, predominantly inattentive type).

##### 2.3.2.2 Final diagnosis

The diagnosis determined during the most recent clinical evaluation, assessed at an average follow-up of 3.5 years.

#### 2.3.3 Stability measures

##### 2.3.3.1 Persistence of ADHD diagnosis

A binary variable indicating whether the ADHD diagnosis persisted or was in remission at the final evaluation.

##### 2.3.3.2 Subtype stability

A categorical variable reflecting whether the initially diagnosed subtype was maintained, changed, or transitioned to another subtype at the follow-up.

#### 2.3.4 Comorbidities

##### 2.3.4.1 Presence of comorbid conditions

We defined comorbid conditions as the presence or absence of anxiety, depression, learning disabilities, trauma, impulse control disorders, substance use disorders (including alcohol, tobacco, cannabis, and behavioral addictions), personality disorders, and physical illnesses or disorders at the time of the evaluation. These diagnoses were established during the initial ADHD evaluation and verified through participants' medical records.

### 2.4 Statistical analysis

A post-hoc power analysis was conducted using G*Power to assess the adequacy of our sample size for detecting significant differences in key outcomes. We aimed for a power level of 0.80 at an alpha level of 0.05. The analysis indicated that our sample of 59 patients was sufficient, with a power of 0.85 for assessing ADHD subtype stability and 0.82 for comparing comorbidities and substance use between the persistent and remission groups. These results confirm the reliability of our findings.

Quantitative variables were expressed as median and interquartile range since they do not follow a normal distribution. The normality of these variables was assessed using the Kolmogorov-Smirnov and Shapiro-Wilk tests. Qualitative variables were expressed as absolute frequencies and percentages. In our analysis, we calculated Cohen's Kappa to measure the agreement between the initial and final diagnoses, with values reflecting the stability of each subtype. This statistic provides insights into diagnostic continuity over the follow-up period. Association presence was evaluated through univariate analysis. For quantitative variables, the Mann-Whitney *U* test was performed, and for qualitative variables, Fisher's exact test or the chi-square test was used. A p-value < 0.05 was considered statistically significant for all tests performed. All analyses were performed using IBM SPSS Statistics for Windows, Version 26.0.

### 2.5 Ethical aspects

This study was conducted following the ethical principles established in the Declaration of Helsinki and has the favorable report of the Chief and Chair of the Psychiatry Department of the Infanta Leonor University Hospital. The rights and well-being of the participants were respected in accordance with applicable ethical and legal standards, and compliance with the provisions of the Data Protection Act was ensured.

## 3 Results

### 3.1 Sample characteristics

59 patients were studied: 45 males (76.3 %) and 14 females (23.7 %) with a mean age of 21.4 years (95 % CI 19.08–23.73) and a median of 19 years. The most common age group at which symptoms began to manifest was 3–6 years (47.5 %), followed by the age group between 7 and 11 years (45.8 %). The most frequent initial presentation was combined ADHD in 35 patients (59.3 %); however, the most prevalent current diagnosis was inattentive ADHD (50.8 %), followed by combined (42.4 %). The majority of the sample (38 patients, 64.4 %) had required pharmacological treatment at some point, and 34 (57.6 %) were still receiving specific treatment for their ADHD at present.

Comorbidities were present in 43 patients (72.9 %). Within these, comorbid psychiatric pathology was present in 34 individuals (57.6 %), with neurodevelopmental disorders (mainly ASD) being the most common (13 patients; 22 %), followed by depressive disorders (10 patients; 16.9 %), and substance use disorder (8 patients; 13.5 %). The majority of individuals in the sample (40 patients; 67.8 %) did not have any kind of substance use. Among those who did, the most common was cannabis use in 7 patients (11.8 %), followed by alcohol in 10.1 % (6 patients).

### 3.2 ADHD stability

Out of the 59 individuals who met DSM-V criteria for ADHD in childhood, 55 continued to meet them in the latest evaluation, representing a persistence rate of 93.2 %.

Of the persistent group, 78.2 % were males, compared to 50 % in the remission group; however, no significant differences were found. In the persistent group, the most frequent age group at which symptoms began was 3–6 years (50.9 %); in the remission group, however, the majority presented symptoms between 7 and 11 years (66.7 %). The median age at the time of their latest evaluation was 19 years for the persistent group and 20.5 years for the remission group.

To further evaluate the distribution of diagnosis persistence by age, current ages were grouped into the following categories: 7–11 years, 12–14 years, 15–17 years, and over 18 years, representing pre-adolescence, early adolescence, late adolescence, and adulthood, respectively. In the persistent group, the majority of individuals (63.6 %) were in the over 18 years age group; for those in the remission group, this percentage was 100 %. However, no statistically significant difference was found between these proportions.

Regarding the years of diagnostic delay, the median in the persistent group was 3 (with an interquartile range of 6), and in the remission group, it was 1 (interquartile range of 0.5). There were no statistically significant differences in terms of age of symptom onset, amount of diagnostic delay, or current age.

For both groups, the most frequent initial subtype was combined (58.2 % for the persistent group and 75 % for the combined group), although the proportion of diagnosis persistence was higher among those who initially presented predominantly inattentive symptoms (95.83 %) than combined (91.42 %). However, these differences were not statistically significant.

The need for treatment was only the most frequent option in the persistent group (34 patients; 61.8 %) compared to 25 % in the remission group (1 patient). There were no differences in terms of treatment need in both groups.

Differences were found in the proportion of individuals who maintained specific follow-up in the specialized ADHD Unit at present (61.8 % in the persistent group vs. 0 % in the remission group; p = 0.0028). The demographic and clinical characteristics of each group are summarized in Table 1.

**Table 1:** Demographic and clinical characteristics of the persistent and remission groups.

	Persistent (N = 55)		Remission (N = 4) P (<0.05)		
	N	%	N	%	
Gender					0.236
Male	43	78.2	2	50	
Age of symptom onset					0.632
0–2	1	1.9	0	0	
3–6	27	50.9	1	33.3	
7–11	25	47.2	2	66.7	
Current age	0.581				
7–11	6	10.9	0	0	
12–14	4	7.3	0	0	
15–17	10	18.2	0	0	
≥ 18	35	63.6	4	100	
	m	IQR	m	IQR	
Diagnostic delay	3	6	1	0.5	0.475
Initial subtype	0.639				
Inattentive	23	41.8	1	25	
Combined	32	58.2	3	75	
Specific follow-up	34	61.8	0	0	0.028*
Treatment	37	67.3	1	25	0.124

m = Median; IRQ = Interquartile range:

* p < 0.005.

### 3.3 Comorbidities and substance use

In the persistent group (55 patients), comorbidities were observed in 72.7 % of individuals (40 patients), with 18 % of the total sample presenting more than one (10 patients). The main comorbidities in this group were neurodevelopmental disorders and somatic disorders, observed in 21.8 % of patients each, followed by depressive disorders in 16.4 % of individuals, and substance use disorder in 14.5 %. In the remission group, 75 % of patients had some comorbidity. In this case, they corresponded to neurodevelopmental disorders, depressive disorders, impulse control disorders, and personality disorders. No significant differences were found between the two groups regarding the presence of comorbidities.

Regarding the presence of substance use, it was only present in 27.3 % of patients in the persistent group. Among those who did use substances, cannabis was the most common substance, observed in 10.9 %, followed by tobacco use in 7.3 %, alcohol use, and behavioral addictions in 5.5 % of patients each. Additionally, one individual presented with multiple substance use. In the remission group, all individuals had some form of substance use, with one patient presenting more than one. In this case, alcohol consumption was the most common (75 %). Significant differences were found in the proportion of patients consuming alcohol (7.3 % in the persistent group vs. 75 % in the remission group; p = 0.002). No significant differences were found in the rest of the variables. The presence of comorbidities, as well as substance use, is summarized in Table 2.

**Table 2:** Comorbidities and substance use in the persistent and remission groups.

	Persistent (N = 55)		Remission (N = 4) P (<0.05)			
	N	%	N	%		
Comorbidity						
	Neurodevelopmental disorder					
		12	21.8	1	25	0.641
	Depressive disorder	9	16.4	1	25	0.534
	Anxiety disorder	3	5.5	0	0	1
	Trauma	1	1.8	0	0	1
	Impulse control disorder	2	3.6	1	25	0.060
	Substance abuse disorder	8	14.5	0	0	0.549
	Alcohol	3	5.5	3	75	0.002*
	Tobacco	4	7.3	1	25	0.305
	Cannabis	6	10.9	1	25	0.405
	Behavioral addiction	3	5.5	0	0	0.807
	Personality disorder	2	3.6	1	25	0.060
	Physical illness/disorder	12	21.8	0	0	0.572

* p < 0.005.

### 3.4 Stability of the initial subtype

**Fig. 1:**
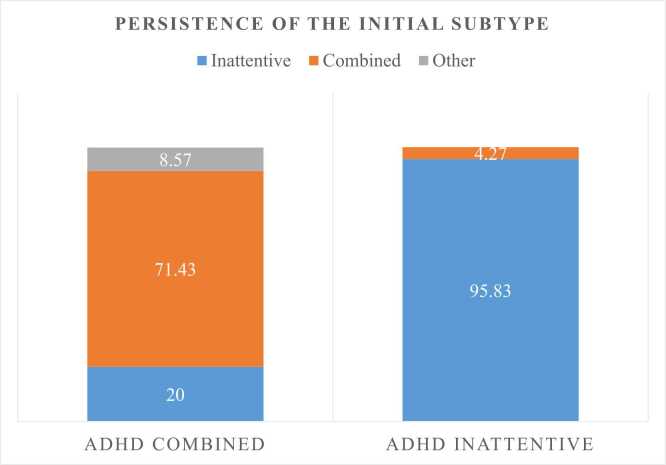
Evolution of each initial presentation.

We examined the stability of the initially diagnosed ADHD subtype over time, focusing on retention rates and transitions. Out of the 59 participants in the study, 81.3 % retained their initially diagnosed subtype. Fig. 1 depicts the evolution of each initial presentation. The most stable subtype was predominantly inattentive, with 95.83 % of individuals initially diagnosed with this subtype continuing to present it in the last evaluation. Among the patients initially diagnosed with predominantly combined ADHD, the majority (71.43 %) retained this subtype.

Among individuals who experienced a change in diagnosis, the most frequent transition was from the combined subtype to the inattentive subtype (20 %), followed by transitions from the combined subtype to ADHD remission (8.57 %) and from inattentive to remission (4.17 %). Notably, no transitions were observed from the inattentive subtype to the combined subtype or from either subtype to predominantly hyperactive-impulsive.

To assess the agreement of the specific diagnosis for each subtype between the first and last evaluation, Cohen's Kappa statistic was applied. Good agreement was obtained, with higher stability observed for the inattentive subtype (k = 0.73) compared to the combined subtype (k = 0.67), both significant at p < 0.001 (Table 3). These results reflect the high stability percentages of each subtype noted earlier.

**Table 3:** Demographic and clinical characteristics of the preserved vs non-preserved initial subtype groups.

	Preserved (N = 48)		Non-preserved (N = 11) P (<0.05)			Cohen’s κ
	N	%	N	%		
Gender						0.712
	Male	37	77.1	8	72.7	
Age of symptom onset						0.051
	0–2	0	0	1	11.1	
	3–6	25	53.2	3	33.3	
	7–11	22	46.8	5	55.6	
Current age						0.352
	7–11	6	12.5	0	0	1*
	12–14	4	8.3	0	0	1*
	15–17	7	14.6	3	27.3	0.44
	≥ 18	31	64.6	8	72.7	0.63*
		m	IRQ	m	IRQ	
Diagnostic delay	6	7	2	3	0.322	
Initial subtype					0.020	
Inattentive	23	47.9	1	9.1		0.73*
Combined	25	52.1	10	90.9		0.67*
Specific follow-up	31	64.6	7	63.6	0.819	
Treatment	25	71.4	13	54.2	0.953	

* p < 0.005; m = Median; IRQ = Interquartile range.

To evaluate the effect of age on diagnosis concordance, Cohen's Kappa statistic was applied across current age subgroups. Perfect concordance was observed in the groups aged 7–11 years and 12–14 years (both p < 0.000). However, in the age group of 15–17 years, poor concordance was observed, with no significant findings. Conversely, in the adult group, good concordance was achieved (k = 0.63), which was statistically significant.

Factors associated with subtype stability were evaluated, revealing significant differences in the stability proportions of each subtype. As mentioned earlier, 95.83 % stability was found in individuals with the initially inattentive subtype, compared to 71.43 % in those with the combined subtype at the beginning (p = 0.02). The group with a stable subtype predominantly presented symptoms between 3 and 6 years in 53.2 % of cases. In contrast, the group that experienced a subtype change more frequently reported symptom onset between 7 and 11 years (55.6 %), with this difference nearing significance (p = 0.051). However, no significant differences were found in other demographic and clinical variables (Table 3).

The median current ages for individuals in the stable and changed subtype groups were 19 years and 20 years, respectively. Additionally, each group experienced median diagnostic delays of 6 years and 2 years, respectively. Table 3 summarizes the demographic and clinical characteristics of the group with a preserved initial subtype compared to the group with a change in initial diagnosis.

### 3.5 Preservation of the initial subtype

Secondly, we explored the characteristics, comorbidities, and addiction patterns among individuals who preserved their initial ADHD subtype compared to those who did not. To further investigate the potential relationship between substance use and subtype stability, we conducted a separate analysis examining the prevalence of substance use in individuals who preserved their initial subtype versus those who did not. This analysis allowed us to isolate the effect of substance use and identify any specific patterns that might be associated with subtype stability or change.

Within the preservation group, a significant proportion of individuals (72.9 %) presented with comorbidities. The most frequently reported comorbid conditions included physical illnesses (22.9 %), neurodevelopmental disorders (20.8 %), and both depressive and substance use disorders (16.7 %). In contrast, among those who did not preserve their initial subtype, comorbidities were similarly prevalent, affecting 72.7 % of this group. However, the distribution of specific comorbidities differed; neurodevelopmental disorders were most common (27.3 %), followed by depressive disorders (18.2 %). Notably, significant differences emerged regarding the proportion of patients with comorbidities linked to traumatic events, with a stark contrast of 0 % in the preservation group compared to 9.1 % in those who changed diagnosis (p = 0.035).

Additionally, the presence of addictions was assessed in both groups. In the preservation group, 31.3 % reported some form of addiction, with cannabis use (12.5 %) being the most prevalent, followed by tobacco (8.3 %) and behavioral addictions (6.3 %). In contrast, 36.4 % of individuals who experienced a change in subtype reported addictions, with alcohol consumption being the most frequently cited (27.3 %). Significant differences were noted in alcohol use, with 6.3 % in the preservation group compared to 27.3 % in the change group (p = 0.037). No significant differences were observed for other variables.

Table 4 summarizes the prevalence of comorbidities and addictions across both groups, highlighting the varying profiles of those who preserved their initial subtype versus those who did not.

**Table 4:** Comorbidities and substance use in the preserved vs non-preserved initial subtype groups.

	Preserved (N = 48)	Non-preserved (N = 11) P (<0.05)			
N	%	N	%		
Comorbidity					
Neurodevelopmental	disorder				
	10	20.8	3	27.3	0.693
Depressive disorder	8	16.7	2	18.2	1
Anxiety disorder	3	6.3	0	0	0.618
Trauma	0	0	1	9.1	0.035*
Impulse control disorder	2	4.2	1	9.1	0.468
Substance abuse disorder	8	16.7	0	0	0.330
Alcohol	3	6.3	3	27.3	0.037*
Tobacco	4	8.3	1	9.1	1
Cannabis	6	12.5	1	9.1	1
Behavioral addiction	3	6.3	0	0	0.618
Personality disorder	2	4.2	1	9.1	0.468
Physical illness/disorder	11	22.9	1	9.1	0.431

* p < 0.005

## 4 Discussion

The stability of ADHD has been a subject of debate and has generated conflicting findings in previous research¹ . Given the high incidence of the disorder and its repercussions on those affected, it is crucial to study its clinical course, especially during the transition from childhood and adolescence to adulthood. Understanding how ADHD symptoms manifest and evolve during these formative years can significantly improve diagnostic and treatment approaches for affected individuals.

This study examined 59 individuals from a specialized ADHD consultation unit, applying DSM-5 diagnostic criteria. Our goal was to evaluate the stability of ADHD, focusing on both the persistence of the diagnosis and transitions between subtypes, along with associated factors. The persistence rate of ADHD in our sample was 93.2 %, which is higher than what many previous studies observed, but consistent with others that applied similar diagnostic criteria.^16^, ^17^, ^18^ This result reflects the greater sensitivity of DSM-5 criteria compared to previous editions, in which there were no specific guidelines for adults. Previous studies have suggested that the low stability percentages they reported were due to the inadequacy of earlier criteria when applied to adults.^19^ Our results support this expectation, showing higher persistence rates than those reported in earlier studies.

### 4.1 ADHD persistence and age

While traditional views suggest that ADHD symptoms attenuate with age, our study found no significant association between current age and disorder persistence. To mitigate potential bias from a wide age range, we analyzed specific age subgroups and compared concordance coefficients. Although a slight decrease in diagnostic concordance was observed in older age groups, persistence remained high across all groups. These findings align with recent research using DSM-5 criteria, which also reported no significant age-related decline in persistence.^20^ This suggests that previous reports of age-related symptom reduction may reflect the insensitivity of earlier diagnostic criteria for adults rather than a genuine decrease in symptoms.^20^

Notably, our study sample was drawn from a clinical setting, where symptoms are typically more severe and persistent compared to community samples. This potential selection bias likely contributes to the observed high persistence rates across all age groups.

### 4.2 Gender and diagnostic stability

No significant difference was found in ADHD persistence between genders, indicating that gender is not a factor influencing diagnostic stability. This result is consistent with those obtained in previous studies.^21^ Additionally, no significant differences were observed in the proportion of years of diagnostic delay or treatment uptake between genders, suggesting these factors are unlikely to affect stability.

### 4.3 The role of comorbidities and follow-up

Although a relationship between comorbidity and disorder persistence was expected, our findings did not significantly support this association. Previous research on this topic has also found no significant differences between these variables. ADHD poses persistent developmental risks across an individual's lifespan, correlating with various mental disorders, educational and occupational setbacks, social impairments, and the development of addictions.^22^ ADHD comorbidities tend to persist, as documented in prior research, regardless of whether ADHD symptoms themselves remit. These findings emphasize the importance of comprehensive follow-up in individuals diagnosed with ADHD, focusing on early detection and treatment of associated disorders.

### 4.4 Substance use and ADHD stability

Contrary to expectations, a higher percentage of addictions was observed in the remissive group compared to the persistent group, although only the association between alcohol consumption and disorder stability reached statistical significance. This finding could be influenced by the higher proportion of individuals in the remissive group who were of substance use age. As suggested in previous studies with similar results, it may also indicate that some consequences of ADHD, such as substance use, persist despite remission of core symptoms.^23^ This further highlights the importance of continuous monitoring in these patients.

### 4.5 ADHD subtypes and stability

A slightly higher proportion of persistent ADHD cases was observed among those with an initially predominantly inattentive presentation (95.83 %) compared to those with an initially combined presentation (91.42 %). However, these differences were not statistically significant, suggesting that the initial presentation form of ADHD likely does not influence long-term persistence. Nevertheless, the association between the initial presentation and subtype stability was significant. Predominantly inattentive ADHD was found to be the most stable, with 93.83 % of individuals retaining this subtype. This may suggest that hyperactivity symptoms tend to attenuate over time, while inattention symptoms persist and become more prominent as individuals age and face increased attentional demands.

A significant relationship was observed between subtype stability and the age of symptom onset. In the group with stable subtypes, most individuals presented symptoms between the ages of 3 and 6, while those who transitioned between subtypes more often reported symptom onset between the ages of 7 and 11. Since all transitions observed involved a reduction in symptoms (either remission or a shift from combined to inattentive subtype), these findings likely reflect that earlier-onset cases represent a more severe form of the disorder.

### 4.6 Transition period considerations

Understanding the stability of ADHD diagnoses during the transition from childhood to adulthood is critical, as this period involves significant developmental changes that can influence symptom manifestation. In this study, ADHD was assessed at two distinct time points: the first visit to the ADHD consultation unit and at an average follow-up of 3.5 years. This longitudinal approach provided insight into how ADHD symptoms may evolve or stabilize over time.

The high rate of persistence (93.2 %) challenges the traditional notion that ADHD symptoms attenuate with age. This suggests that ADHD remains a significant concern during key life transitions, such as entering adolescence or adulthood, when academic and social demands increase. Consequently, clinicians should prioritize the ongoing evaluation of symptoms and the effectiveness of treatments during these transitions to ensure patients receive the necessary support to address their evolving needs.

Additionally, the transitions observed between ADHD subtypes, particularly from combined to predominantly inattentive forms, highlight the dynamic nature of the disorder. Such changes suggest that clinicians may need to adjust treatment approaches over time. For instance, while stimulant medications may effectively manage hyperactivity, they may be less effective for inattention, requiring careful monitoring and potential therapeutic modifications.^24^

## 5 Limitations

Several limitations should be considered when interpreting our results. First, the relatively small sample size may limit the generalizability of our findings. Moreover, the wide age range among participants could have affected patterns of substance use and other outcomes. For example, younger individuals, particularly those in the persistence group, may have different levels of exposure to substance use compared to older individuals in the remissive group. The age disparity should be considered when interpreting the relationship between substance use and subtype stability.

Additionally, some comparisons included very few participants, raising concerns about the robustness of our findings. For example, the higher prevalence of alcohol use in the remissive group compared to the persistent group could be influenced by the smaller sample size in the persistent group.

Moreover, the sample was drawn from a specialized ADHD clinic, which may represent individuals with more severe ADHD than in the general population, potentially limiting the generalizability of our findings to individuals with less severe forms of the disorder.

## 6 Research implications

To overcome the limitations of the current study, future research should prioritize enrolling a larger and more diverse sample. This would not only enhance the generalizability of the findings but also facilitate more nuanced analyses of factors such as age, substance use, and subtype stability across different demographics. Additionally, employing advanced statistical methods to control for age-related effects on substance use patterns would be beneficial. Given the significant influence of age on ADHD and substance use, accounting for age-related variables will provide a clearer understanding of the true relationship between ADHD and substance use over time.

Furthermore, increasing the sample size in comparison groups is essential to strengthen the reliability of findings and reduce bias. A larger sample would improve statistical power, allowing for the detection of meaningful differences between groups. Evenmore, future studies should include individuals with varying levels of ADHD severity to ensure that the results are more representative of the broader ADHD population, reflecting the experiences of individuals with both mild and severe manifestations of the disorder. Lastly, although studies evaluating the challenges of transitioning to adulthood in individuals with ADHD already exist, it would be valuable to directly explore the opinions, beliefs, fears, and attitudes of patients with ADHD as they navigate this transition through healthcare systems that, in most regions, are markedly different. Understanding how ADHD patients manage their diagnoses and treatment across different healthcare systems will enhance our understanding of the factors affecting diagnostic stability and persistence, as well as highlight potential barriers they face during this crucial life stage.

By addressing these recommendations, future research can provide a more comprehensive understanding of ADHD persistence and its relationship with co-occurring conditions. This will ultimately help inform better diagnostic and treatment strategies for individuals across a range of ages and severity levels.

## 7 Practice implications

The practical implications of this study underscore the importance of ongoing and personalized care in managing ADHD, especially during critical life transitions like adolescence and adulthood. Given the high persistence rate of ADHD observed (93.2 %), clinicians should not assume that symptom attenuation with age negates the need for continued treatment and monitoring. The dynamic nature of ADHD, including the transitions between subtypes, suggests that treatment plans may require adjustment over time to address evolving symptom profiles. Importantly, the study highlights the need for comprehensive follow-up, focusing on the early detection and management of associated disorders, such as substance use, which may persist even when core ADHD symptoms remit. This proactive approach can help mitigate the long-term risks of comorbidities and improve overall patient outcomes.

## 8 Conclusions

In our sample ADHD shows a high persistence rate from childhood to adulthood. The stability of ADHD was consistent across different age groups and genders, suggesting that factors like age and gender do not significantly influence disorder persistence. ADHD management does not end with symptom remission. Ongoing care and monitoring, especially focusing on the risks of substance use, should be embedded into practice to prevent long-term negative outcomes.

## Funding

This research did not receive any specific grant from funding agencies in the public, commercial, or not-for-profit sectors.

## Ethical Statement

All procedures were performed in compliance with relevant laws and institutional guidelines and have been approved by the appropriate institutional committee. This study was conducted following the ethical principles established in the Declaration of Helsinki and has the favorable report of the Chief and Chair of the Psychiatry Department of the Infanta Leonor University Hospital. The rights and well-being of the participants were respected in accordance with applicable ethical and legal standards, and compliance with the provisions of the Data Protection Act was ensured.

## CRediT authorship contribution statement

**Javier Quintero:** Writing – review & editing, Validation, Supervision, Methodology, Investigation, Conceptualization. **Fernando Mora Mínguez:** Writing – review & editing, Supervision, Methodology, Investigation, Formal analysis, Conceptualization. **Miguel Ángel Álvarez-Mon:** Writing – review & editing, Project administration, Methodology, Investigation, Formal analysis. **Cristina Bonilla Sanz:** Writing – review & editing, Writing – original draft, Methodology, Investigation, Formal analysis, Data curation, Conceptualization. **Alberto Rodríguez-Quiroga:** Writing – review & editing, Writing – original draft, Methodology, Investigation, Formal analysis, Data curation.

## Declaration of Competing Interest

The authors declare that they have no known competing financial interests or personal relationships that could have appeared to influence the work reported in this paper.

## Data Availability

Data will be made available on request.
